# Imrecoxib attenuates osteoarthritis by modulating synovial macrophage polarization through inactivating COX-2/PGE2 signaling pathway

**DOI:** 10.3389/fbioe.2025.1526092

**Published:** 2025-04-28

**Authors:** Peng Peng, Wanling Zheng, Yuchen Liu, Jingyuan Huang, Bin Zhang, Jiawei Shen, Jiangang Cao

**Affiliations:** ^1^ Department of Sports injury and Arthroscopy, Tianjin University Tianjin Hospital, Tianjin, China; ^2^ Department of Neurosurgery, The Second Affiliated Hospital of Xuzhou Medical University, Xuzhou, China; ^3^ Department of Orthopedics, Xinhua Hospital, Shanghai Jiao Tong University School of Medicine, Shanghai, China; ^4^ Department of Dermatology and cosmetology, Minhang Hospital, Fudan University, Shanghai, China; ^5^ Department of Hand Surgery, Huashan Hospital, Fudan University, Shanghai, China; ^6^ International Science and Technology Cooperation Base of Spinal Cord Injury, Tianjin Key Laboratory of Spine and Spinal Cord Injury, Department of Orthopedics, Tianjin Medical University General Hospital, Tianjin, China

**Keywords:** osteoarthritis, imrecoxib, macrophage polarization, cartilage protection, COX 2/PGE2

## Abstract

**Introduction:**

Although biomaterials strategies have been regarded as a promising approach for the treatment of osteoarthritis (OA), identifying novel drugs to be delivered for modulate macrophage polarization is still unclear. As a commonly used non-steroidal anti-inflammatory drug for OA, Imrecoxib may be a novel drug to direct and sustain macrophage phenotype. However, the specific protective mechanism of Imrecoxib in OA remains unclear. This study aims to investigate whether Imrecoxib would treat OA by regulating synovial macrophage polarization.

**Methods:**

The research involves constructing mouse destabilization of medial meniscus (DMM) model to assess the changes in pain, bone destruction, cartilage degeneration, and synovial macrophage phenotypes following Imrecoxib treatment. Additionally, the effects of macrophage conditioned medium (CM) pretreated with Imrecoxib on the chondrocyte apoptosis, inflammation and degeneration-related factor expression were evaluated. The role of COX-2/PGE2 signaling pathway in the macrophage phenotype changes was further investigated.

**Results:**

We found that Imrecoxib alleviated pain, cartilage degeneration and synovitis, promoted polarization of M1 macrophages toward M2 phenotype *in vivo* and *in vitro*. *In vitro* experiments, Imrecoxib-CM protected chondrocyte by modulating macrophage polarization. Furthermore, Imrecoxib regulates macrophage polarization through the COX-2/PGE2 pathway.

**Conclusion:**

This study unravels that Imrecoxib protects joint cartilage and attenuates osteoarthritis by modulating synovial macrophage polarization through inactivating COX-2/PGE2 signaling pathway, providing new drug delivery strategy for the clinical treatment of OA.

## 1 Introduction

Osteoarthritis (OA) is a common musculoskeletal disease, leading to joint pain, deformity, and restricted mobility (Liu et al., 2023; Li et al., 2020a). Studies indicate that the incidence of OA in individuals aged 65 and above exceeds 75%, and it is anticipated to become a leading cause of disability worldwide by the year 2030 (Thomas et al., 2014; Hunter and Bierma-Zeinstra, 2019). The primary characteristic changes in OA include degeneration of joint cartilage, narrowing of joint spaces, formation of bone spurs, reshaping of subchondral bone and synovial inflammation (Hügle and Geurts, 2017). Epidemiological surveys reveal that approximately 89% of knee OA patients exhibit severe synovitis, which plays a crucial role in the occurrence and development of OA (Scanzello and Goldring, 2012).

Macrophage cell-based therapies represent a promising approach for the treatment of inflammatory diseases, owing to their intrinsic ability to modulate the immune microenvironment and orchestrate tissue responses (Roemer et al., 2010; Lee et al., 2024). Activated macrophages exhibit two main phenotypes, namely, M1 (classically activated) and M2 (alternatively activated) types. M1 macrophages primarily secrete pro-inflammatory factors, such as IL-1β, IL-6 and TNF-α, resulting in cartilage degeneration. However, M2 macrophages secrete IL-10, Arg-1, and TGF-β, which plays key roles in suppressing inflammation and promoting tissue repair and regeneration (Wang et al., 2023). In the synovium of OA patients, there is a significant increase in the proportion of M1 macrophages, while M2 macrophages are relatively scarce (Sun et al., 2020). Studies indicate that when synovial macrophages primarily polarize towards M1 type, whether in collagenase-induced or traumatic OA models, the severity of OA is exacerbated (Liu et al., 2018). Therefore, inhibiting the polarization of M1 macrophages and promoting the conversion of M1 to M2 may represent a novel strategy for treating OA (Manferdini et al., 2017).

A substantial body of research indicates that biomaterials strategies to modulate macrophage polarization have been regarded as a promising approach for the treatment of osteoarthritis (OA) (Lee et al., 2024). Biomaterials are macromolecular/supramolecular systems designed to dynamically interface with biological environments, focusing on biocompatibility, spatiotemporal control, and multifunctionality. Examples include hydrogels, ECM-mimetic scaffolds, or membrane-coated nanoparticles (Li et al., 2023). Leveraging their inherent immunomodulatory properties and inflammation-homing capability, nanoghosts (NGs) were endocytosed by chondrocytes and localized to lysosomes, subsequently downregulating the expression of COX-2 and PGE2 at both mRNA and protein levels, showing significantly greater efficacy compared to the untreated group (D'Atri et al., 2021). Additionally, artificial M2 macrophages (AM2M) engineered with M2 macrophage membrane shells not only blocked interleukin-induced acute inflammatory injury but also circumvented immune stimulation triggered by chondroitin sulfate (ChS) (Ma et al., 2021). However, current bionic biomaterials for OA still lack comprehensive *in vivo* and *in vitro* studies to clarify their mechanistic actions and long-term therapeutic effects, hindering their clinical translation (Li et al., 2019).

**FIGURE 1:**
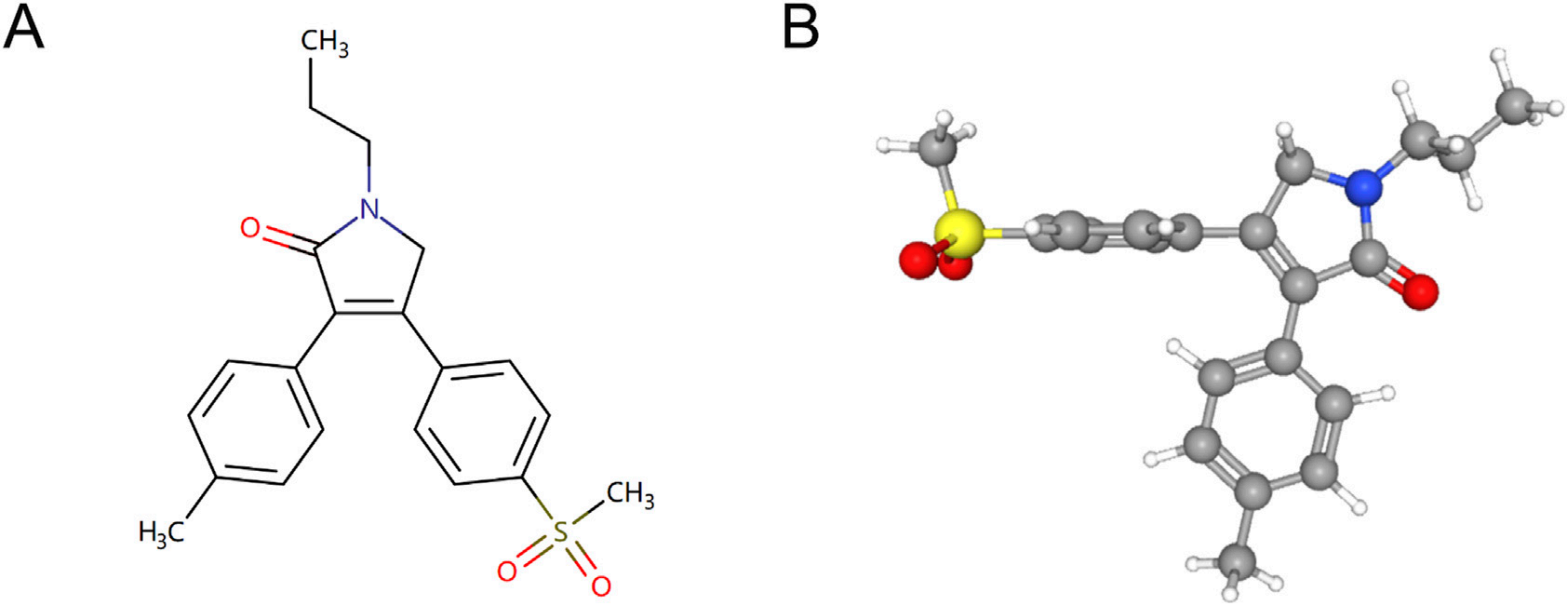
Chemical structures of Imrecoxib. **(A)** 2D structure of Imrecoxib. **(B)** 3D structure of Imrecoxib.

In contrast, small-molecule synthetic compounds directly modulate pathological targets, as a commonly used non-steroidal anti-inflammatory drugs for OA, Imrecoxib may be a novel drug to direct and sustain macrophage phenotype (Wang et al., 2024) (Figures 1A,B). More importantly, macrophages could identify apoptotic cells through the COX-2/PGE2 pathway, inducing the production of inflammatory or anti-inflammatory factors. Hence, targeting the COX-2/PGE2 signaling pathway can mediate the phenotypic transformation and function of wound macrophages (Xia et al., 2018). Imrecoxib, a selective inhibitor of the COX-2/PGE2 pathway, is one of the commonly used nonsteroidal anti-inflammatory drugs for clinical treatment of OA (Chen et al., 2004). Numerous studies have suggested that NSAIDs can alleviate joint inflammation and delay cartilage degradation (Nakata et al., 2018; Zeng et al., 2015; Yang et al., 2018). However, there is currently no research indicating whether Imrecoxib exerts its anti-inflammatory effects by regulating the phenotypic polarization of synovial macrophages.

Given the crucial role of synovial macrophages in OA, the mechanism by which Imrecoxib protects joint cartilage and treats OA through the regulation of macrophage polarization deserves further exploration. Our study demonstrates that Imrecoxib protects joint cartilage and attenuates osteoarthritis by modulating synovial macrophage polarization through inactivating COX-2/PGE2 signaling pathway, providing new drug delivery strategy for the clinical treatment of OA.

## 2 Materials and methods

### 2.1 Experimental animals

In this experiment, mice were housed under a constant temperature and humidity environment with regular 12-hour light/dark cycles as well as free access to a standard diet and water. All experimental procedures were approved by the Ethics Committee of Institute of Radiation Medicine Chinese Academy of Medical Sciences (approval number: IRM-DWLL-2018010).

### 2.2 Osteoarthritis model and treatment

OA was established in 10-week-old C57BL/6 mice by destabilization of the medial meniscus (DMM) of the right knee (Li et al., 2020a). Briefly, after anesthesia with 1.5% tribromoethanol 200 mg/kg body weight i.p. injection, surgery was completed by transection of the anteromedial meniscotibial ligament and the medial collateral ligament. They were randomly divided into the following groups: the control group, DMM group, DMM + low-dose Imrecoxib group (5 mg/ml/day), DMM + medium-dose Imrecoxib group (10 mg/ml/day), and DMM + high-dose Imrecoxib group (20 mg/m1/day), with 15 mice in each group. The day after surgery, the mice received Imrecoxib by oral gavage once a day for 12 weeks. We performed histological analysis and graded articular cartilage degeneration using the Osteoarthritis Research Society International (OARSI) guidelines.

### 2.3 Behavioral assessment

Behavioral assessments including mechanical allodynia and thermal hyperalgesia were conducted using von Frey filaments and radiant heat tests, respectively, at pre-surgery and 1, 4, 8 and 12 weeks post-operation.

Mechanical Allodynia (von Frey test): Animals were acclimated in a mesh-bottomed cage for 3 h prior to testing. A graded series of von Frey filaments (forces: 2.44–4.74 g) were applied perpendicularly to the mid-plantar surface of the right hind paw. The minimal force eliciting a positive response (paw withdrawal or licking) was recorded using an up-down paradigm, with termination criteria defined as either five consecutive negative responses or four consecutive positive responses.

Thermal Hyperalgesia (Hargreaves' test): Mice were habituated in a transparent acrylic chamber mounted on a temperature-controlled glass plate (30°C) for 30 min. A focused radiant heat source (5 × 5 mm aperture) was directed to the plantar surface, and the paw withdrawal thermal latency (PWTL) was measured as the time from heat onset to withdrawal. A 20-s cutoff was implemented to prevent tissue injury. Three trials per animal were averaged with 6–8-minute inter-trial intervals.

### 2.4 Immunohistochemistry (IHC) and immunofluorescence (IF)

Specimens were prepared as described previously. The fixed knee joints were decalcified for 30 days using a 10% EDTA solution. Subsequently, the specimens were subjected to dehydration, embedded in paraffin, and serially sectioned at a thickness of 5 μm to ensure the inclusion of the entire joint. For IHC analysis, sections were stained with primary antibodies: IL-6 (1:100, ab290735), TNF-α (1:100, ab183218), MMP3 (1:100, ab52915), IL-1β (1:100, ab315084). For immunofluorescence, sections were stained with primary antibodies: CD86 (1:100, Servicebio GB13585), CD206 (1:100, Servicebio GB11349) and FITC-labeled or CY3-labeled secondary antibodies (1:200, Servicebio GB22303; 1:200, Servicebio GB21303). The sections were mounted with medium containing DAPI and images were obtained using a fluorescence microscope (Nikon Eclipse Ti-SR). Three sagittal sections containing the lesion were selected for each group. In each section, four distinct areas within the lesion were chosen, photographed, and analyzed. The polarization of macrophages was then quantified using ImageJ software.

### 2.5 Micro-CT

The entire fixed joint was subjected to Micro CT scanning (μCT 40; Scanco, Zurich, Switzerland) as to assess bone damage and repair. The scanning precision was 8.96 μm, with a filter selection of 0.5 mL Al and a 180° helical scan. The scan voltage and current were set at 60 kV and 368 μA, respectively. NRecon software was used for data reconstruction, and Data Viewer software was employed to observe the bone structure in coronal, sagittal, and transverse planes. CTan software was utilized for 3D quantitative analysis, and CTvox/CTvol software was employed to generate 3D effect images. The analysis primarily focused on bone trabecular data at the distal end of the femur and the tibial plateau. The ROI for trabecular bone at the distal end of the femur was selected within the range of 0.215 mm–1.94 mm from the growth plate. Key evaluation parameters included the ratio of trabecular bone volume to total bone volume (BV/TV), trabecular separation (Th.Sp), trabecular number (Tb.N), and trabecular thickness (Tb.Th).

### 2.6 Macrophage repolarization from M1 to M2 phenotype

Macrophages (10^6^ cells per well) seeded in a 6-well plate were stimulated with or without LPS (Sigma-Aldrich, 100 ng/mL) and various concentrations of Imrecoxib for 24 h. The experiment was divided into five groups: the macrophage group, M1-type macrophage group, M1 + low-dose Imrecoxib group (10 μmol/L), M1 + medium-dose Imrecoxib group (50 μmol/L), and M1 + high-dose Imrecoxib group (100 μmol/L). The qRT-PCR and immunofluorescene were conducted to detect the polarization transitions.

### 2.7 Macrophage polarization induction and collection of conditioned medium

On the sixth day, when changing the culture medium, add 100 ng/mL of LPS to the existing culture medium. After 24 h of stimulation, RAW264.7 macrophages (Procell Life Science&Technology Co) polarized into M1 macrophages. Then, the new medium was changed to exclude the effect of residual LPS. The conditioned medium (CM) from macrophages was collected within 24 h of M1 macrophages. Centrifuge the medium at 1000 g for 5 min to remove cell debris and store the supernatant at −80°C for further experiments. The CM was diluted with serum-free culture medium at a 1:1 ratio and added to chondrocytes (Yi et al., 2024). The qRT-PCR and immunofluorescene were conducted to verify the protective role of Imrecoxib in chondrocyte.

### 2.8 Flow cytometry for chondrocyte apoptosis evaluation

Chondrocytes seeded in 6-well plates were stimulated with or without IL-1β (PeproTech, 10 ng/mL) and various concentrations of conditioned medium (CM) for 24 h. The rates of chondrocytes apoptosis were determined by flow cytometry analysis with an Annexin V-FITC/PI Apoptosis kit according to the manufacturer’s instructions (BD Biosciences).

### 2.9 RNA extraction and quantitative real-time PCR (qRT-PCR)

Total RNA from primary chondrocytes and macrophages in different groups was isolated by the Total RNA kit (R6834–01, Omega). Total RNA integrity and quantity were determined using an Agilent 2,100 Bioanalyser (Agilent Technologies, San Jose, CA, USA). Only RNA with an A260/A280 ratio between 1.8 and 2.1 was used. and cDNA was transcribed reversely from total RNA. Reverse transcription was conducted using Transcription High Fidelity cDNA Synthesis Kit (5081963001, Roche) according to manufacturer’s instructions. Finally, we used the LightCycler^®^ 96 system to analyze the mRNA expression levels in cells. The primers used are listed in Table 1. The qRT-PCR conditions were: 95°C for 10 min, 40 cycles of 95°C for 10 s, 60°C for 20 s and 72°C for 15 s. The expression levels of each target gene were calculated with the 2^−ΔΔCT^ method and normalized to the internal control (GAPDH).

**TABLE 1:** The primers of genes used in the RT-qPCR.

Gene	Primer
*Gapdh*-F	5ʹ-CTT​CAT​TGA​CCT​CAA​CTA​CAT​GGT​CTA-3ʹ
*Gapdh*-R	5ʹ-GATGA CAAGCTTCCC ATTCTCAG-3ʹ
*Il-6*-F	5ʹ-CAA​CGA​TGA​TGC​ACT​TGC​AGA-3ʹ
*Il-6*-R	5ʹ-TGT​GAC​TCC​AGC​TTA​TCT​CTT​GG-3ʹ
*Il-1β*-F	5ʹ- TTC​AAG​GGG​ACA​TTA​GGC​AG-3ʹ
*Il-1β*-R	5ʹ-TGT​GCT​GGT​GCT​TCA​TTC​AT-3ʹ
*Tnf-α*-F	5ʹ- CTC​AGC​GAG​GAC​AGC​AAG​G-3ʹ
*Tnf-α*-R	5ʹ-AGG​GAC​AGA​ACC​TGC​CTG​G-3ʹ
*iNos*-F	5ʹ-GCG​CTC​TAG​TGA​AGC​AAA​GC-3ʹ
*iNos*-R	5ʹ-AGT​GAA​ATC​CGA​TGT​GGC​CT-3ʹ
*Arg-1*-F	5ʹ-AGG​CGC​TGT​CAT​CGA​TTT​CT-3ʹ
*Arg-1*-R	5ʹ-TGG​AGT​CCA​GCA​GAC​TCA​AT-3ʹ
*Cd206*-F	5ʹ-CTC​TGT​TCA​GCT​ATT​GGA​CGC-3ʹ
*Cd206*-R	5ʹ-CGG​AAT​TTC​TGG​GAT​TCA​GCT​TC-3ʹ
*Cox-2*-F	5ʹ-CTT​ACA​ATG​CTG​ACT​ATG​GCT​AC-3ʹ
*Cox-2*-R	5ʹ-CTA​CAA​CAC​GGC​ACA​CGA​CT-3ʹ
*Mmp3-*F	5ʹ-TCA​TGA​ACT​TGG​CCA​CTC​CC-3ʹ
*Mmp3-*R	5ʹ-GAA​CAA​GAC​TTC​TCC​CCG​CA-3ʹ
*Adamts5*-F	5ʹ-GGC​ATC​ATT​CAT​GTG​ACA​CC-3ʹ
*Adamts5*-R	5ʹ-CGA​GTA​CTC​AGG​CCC​AAA​TG-3ʹ

### 2.10 Cytokine PGE2 measurements

Pre-polarized RAW264.7 macrophages were treated with various concentrations of Imrecoxib for 24 h. The CM of macrophages was collected, and the concentrations of PGE2 were measured via ELISA kits according to the manufacturer’s guidelines (Enzyme-linked Biotechnology, ml028719).

### 2.11 Statistical analysis

Graphad Pro 8.0 was used for data analysis. The data were shown in mean ± standard error of mean (SEM). One-way ANOVA with Tukey’s *post hoc* test and two-way ANOVA with Bonferroni’s *post hoc* test were used for comparisons among multiple groups. P < 0.05 was considered statistically significant.

## 3 Results

### 3.1 Imrecoxib reduces pain and bone destruction induced by DMM

**FIGURE 2:**
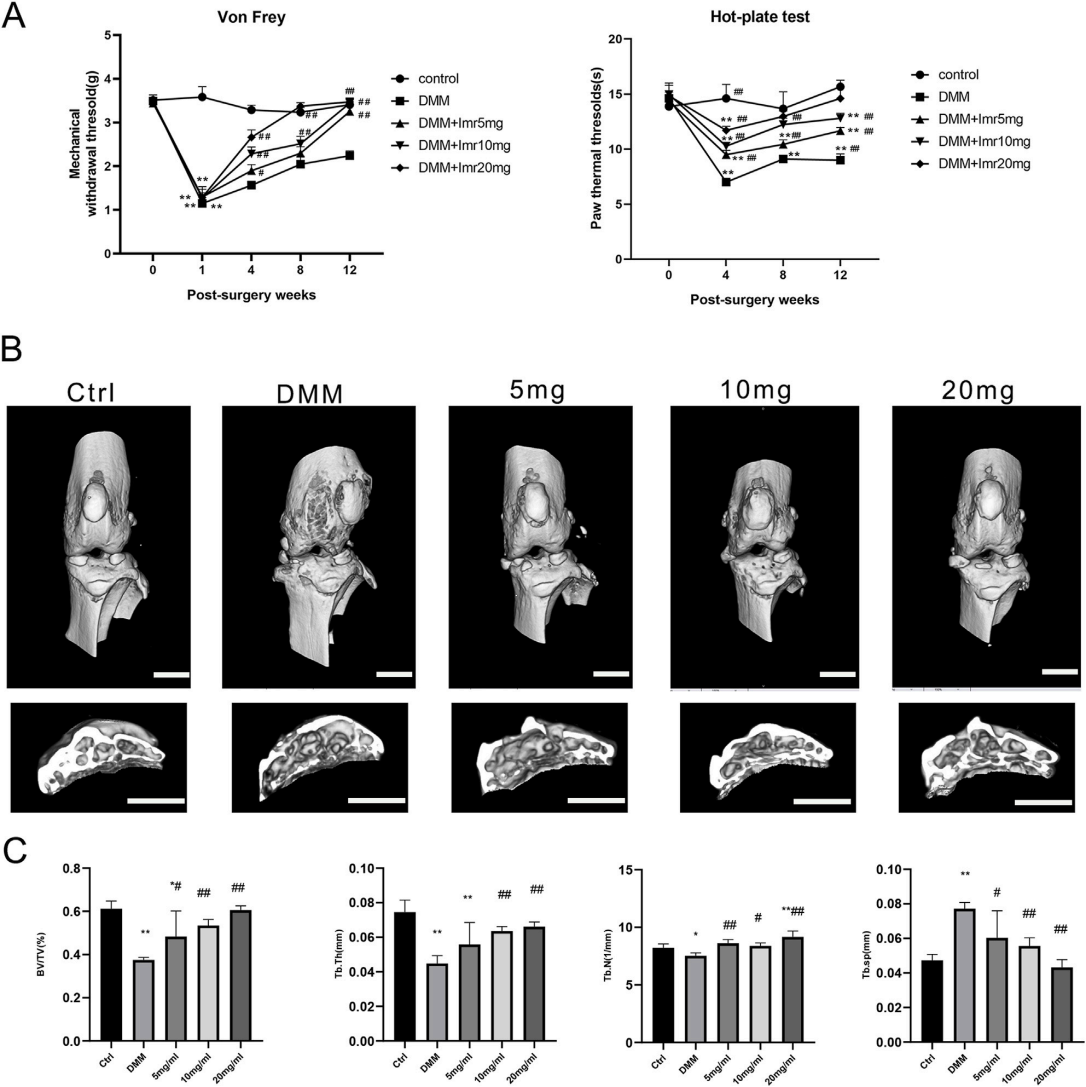
Imrecoxib Reduces Pain and Bone Destruction Induced by DMM. **(A)** The Von Frey test and hot plate test. **(B)** The three-dimensional μCT images of frontal views and cross-section of subchondral bone of the right knee joints. **(C)** Quantitative analysis of BV, BV/TV, Tb.Sp, Tb.N and Tb.Th. *p < 0.05, **p < 0.01 compared to the control group; ^#^p < 0.05, ^##^p < 0.01 compared with the DMM group. Scal bar = 1 mm.

To investigate the sensitivity of mice to noxious stimulation after DMM surgery, the Von Frey and hot plate test was conducted. The results showed no significant differences in baseline paw withdrawal thresholds among the groups before surgery. From the 4th week postoperatively to the 12th week, the paw withdrawal thresholds in the DMM group began to slowly rise and were significantly lower than those in the Imrecoxib group at each time point (p < 0.01), indicating that Imrecoxib alleviates the pain caused by DMM surgery (Figure 2A).

Then, the mouse knee joints were subjected to micro-CT scanning to explore the impact of Imrecoxib on subchondral bone remodeling in OA. In the DMM group, the BV of subchondral bone beneath the tibial plateau was significantly decreased compared to the control group (p < 0.05). Nevertheless, mice treated with Imrecoxib at concentrations of 5, 10, and 20 mg/mL exhibited suppressed bone destruction, as evidenced by BV/TV, Tb.N, Tb.Th, and Tb.Sp (Figures 2B,C). The results indicated that administration of Imrecoxib in a dose-dependent manner significantly reduced bone loss, demonstrating increased integrity of knee joint bones and enhanced subchondral bone mass.

### 3.2 Imrecoxib protects cartilage and delays the progression of OA

In the control group, the articular cartilage surface was smooth and intact, with a well-maintained cartilage structure and tidemark. In contrast, the articular cartilage staining in the DMM group revealed a significant reduction in thickness of articular cartilage and disorganized arrangement. OARSI scores indicated a significant increase in the DMM group compared to the blank control group (p < 0.01). Compared to the DMM group, the Imrecoxib treatment group showed a significant increase in cartilage matrix and joint thickness, with a dose-dependent decrease in OARSI scores (low dose: 5.33 ± 1.15; medium dose: 4.67 ± 1.15; high dose: 2.67 ± 1.15) (p < 0.05).

**FIGURE 3:**
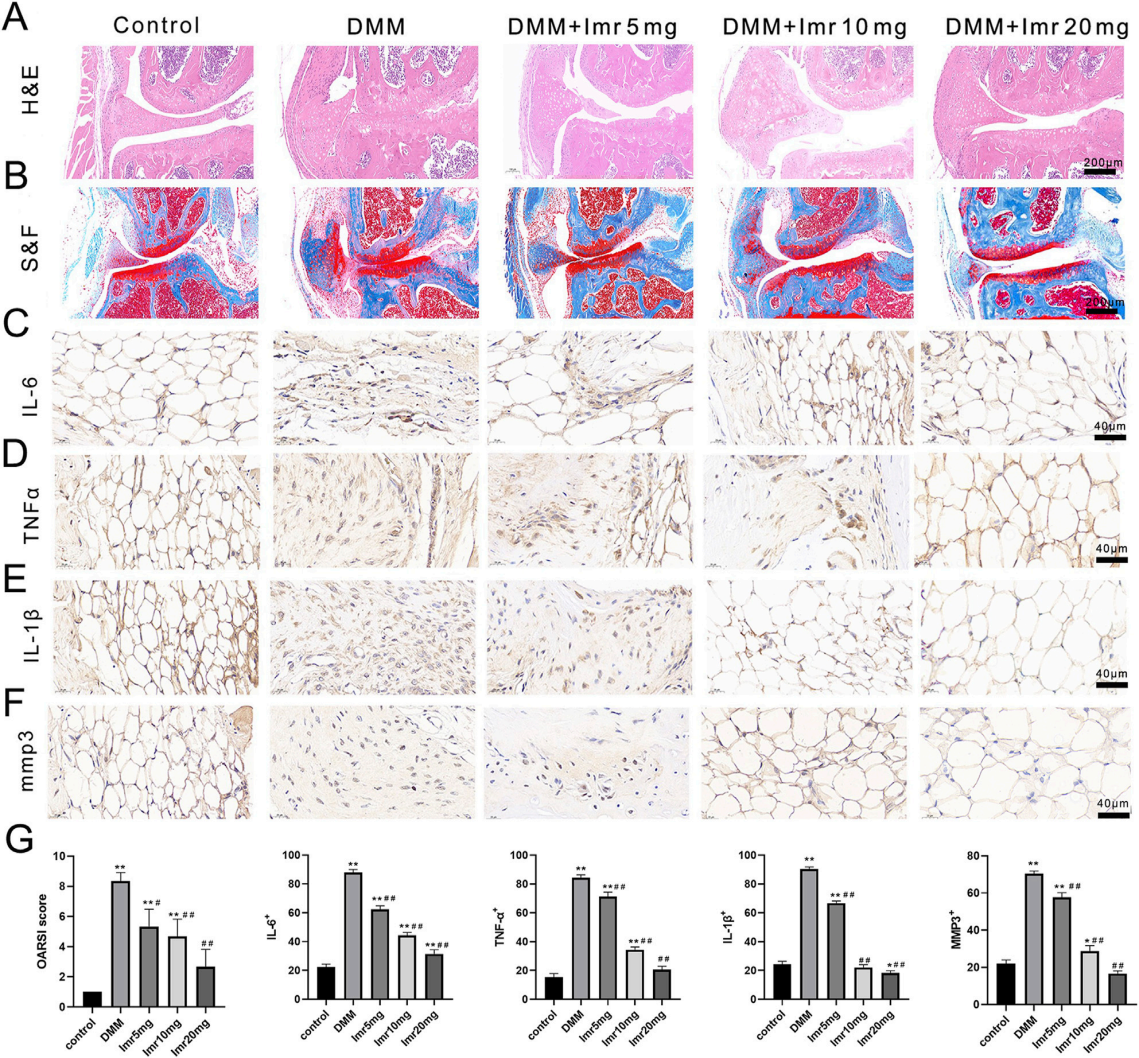
Imrecoxib protects cartilage and delays the progression of OA. **(A, B)** HE and Safranin O-Fast green staining of synovial and articular surfaces of cartilage. IL-6 **(C)**, TNF-α **(D)**, MMP3 **(E)**, and IL-1β **(F)** immunohistochemistry of knee joint medial compartment cartilage. **(G)** Quantitative analysis of OARSI score, IL-6, TNF-α, MMP3, and IL-1β. *p < 0.05, **p < 0.01 compared to the control group; ^#^p < 0.05, ^##^p < 0.01 compared with the DMM group.

Immunohistochemical staining results demonstrated that, compared to the control group, the inflammatory factors IL-6, TNF-α, MMP3, and IL-1β significantly increased in the synovial tissue of the DMM group (p < 0.01). In comparison, the Imrecoxib treatment group exhibited a significant reduction in inflammatory reactions (p < 0.05). In summary, Imrecoxib could protect cartilage and delay the progression of OA (Figure 3).

### 3.3 Imrecoxib promotes synovial macrophage polarization from M1 to M2 phenotype

**FIGURE 4:**
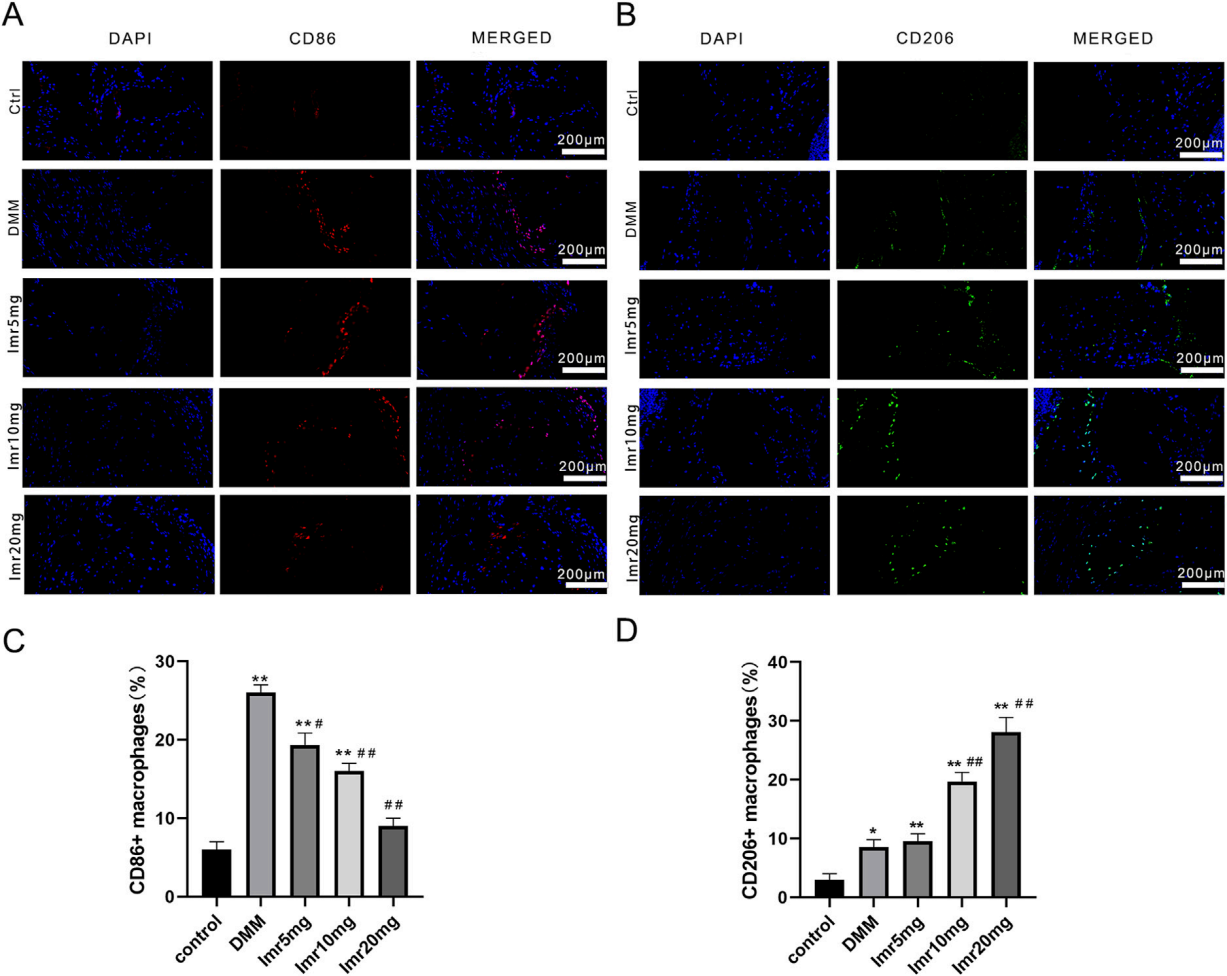
Imrecoxib promotes synovial macrophage polarization from M1 to M2. Fluorescent staining shows changes in **(A)** M1 macrophage markers and **(B)** M2 macrophage markers in synovial tissue. Quantitative analysis of **(C)** CD86^+^ and **(D)** CD206+ cells rates. *p < 0.05, **p < 0.01 compared to the control group; ^#^p < 0.05, ^##^p < 0.01 compared with the DMM group.

Immunofluorescence staining of synovial macrophages revealed that the proportions of CD86^+^ and CD206+ cells in the DMM group were 26.27% ± 0.64% and 8.53% ± 1.29%, respectively. Compared to the DMM group, the Imrecoxib treatment group showed a significant decrease in the proportion of CD86^+^ cells (low dose: 19.50% ± 1.50%; medium dose: 16.10% ± 0.85%; high dose: 9.07% ± 1.90%), while the proportion of CD206+ cells was significantly upregulated (low dose: 9.50% ± 1.32%; medium dose: 19.70% ± 1.54%; high dose: 28.07% ± 2.53%) (Figure 4). These results indicate that Imrecoxib in OA mice can reduce the proportion of M1-type synovial macrophages, increase the proportion of M2-type synovial macrophages.

### 3.4 Imrecoxib regulates macrophages polarization *in vitro*

**FIGURE 5:**
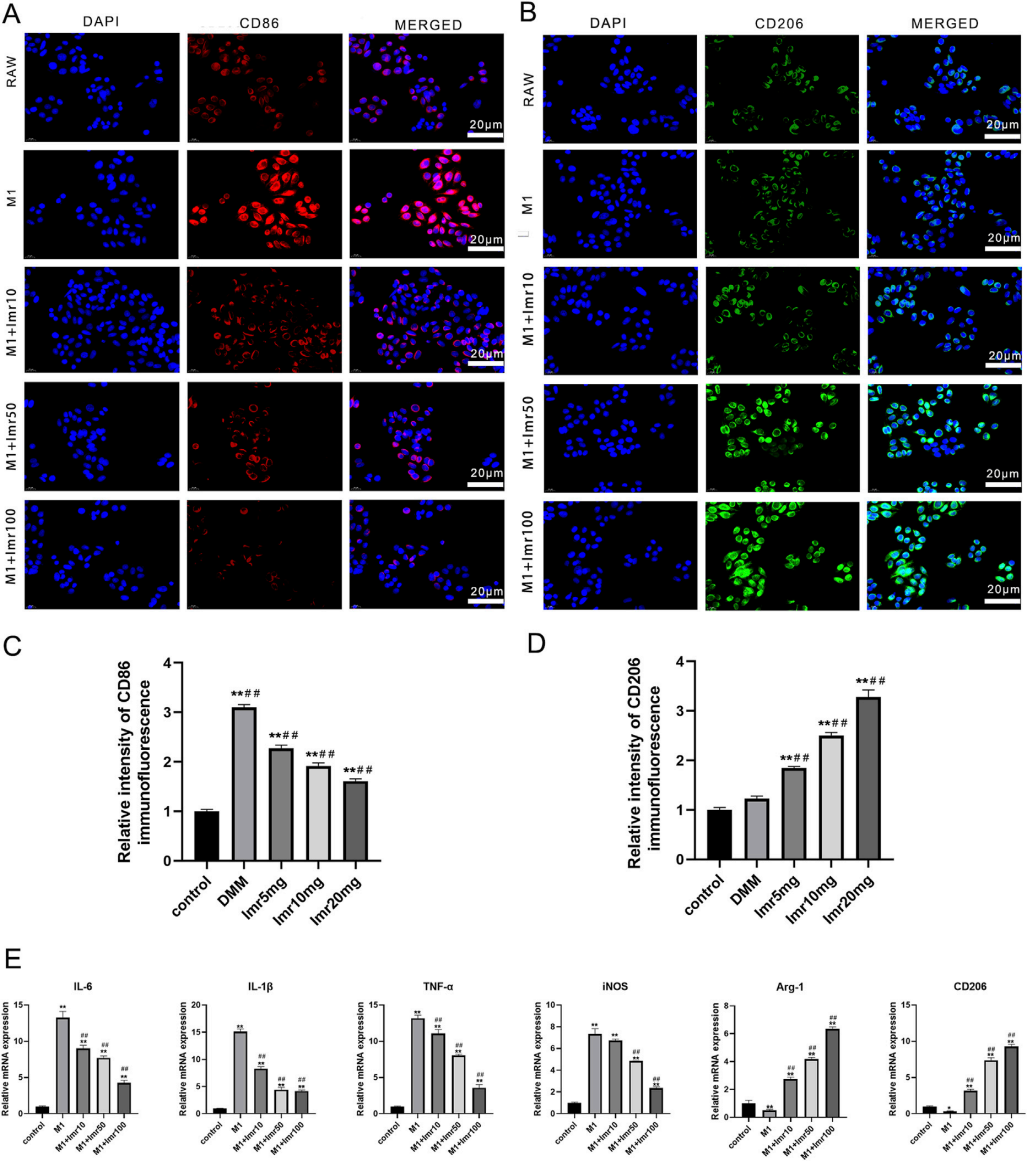
Imrecoxib regulates macrophages polarization *in vitro.* Immunofluorescence staining of **(A)** CD86 and **(B)** CD206 in RAW264.7 macrophages. Quantitative analysis of **(C)** CD86 and **(D)** CD206 immunofluorescence intensity. **(E)** qRT-PCR analysis of M1-related and M2-related markers. *p < 0.05, **p < 0.01 compared to the control group; ^#^p < 0.05, ^##^p < 0.01 compared with the M1 group.

Moreover, Immunofluorescence revealed that Imrecoxib inhibited CD86^+^ cells (M1 phenotype) and promoted the expression of CD206+ cells (M2 phenotype) (Figures 5A–D). Imrecoxib exhibited a dose-dependent effect on RAW264.7 macrophages polarization, which reduced the expression of M1-related genes, including IL-6, IL-1β, TNF-α, IL-12, and iNOS, while increasing the expression of M2-related genes, such as Arg-1 and CD206. These differences were statistically significant (p < 0.01) (Figure 5E). These results indicate that Imrecoxib promotes polarization of M1 macrophages toward M2 phenotype and suppresses the inflammatory response of synovial macrophages.

### 3.5 Imrecoxib-treated macrophage CM prevents impairment of chondrocytes

**FIGURE 6:**
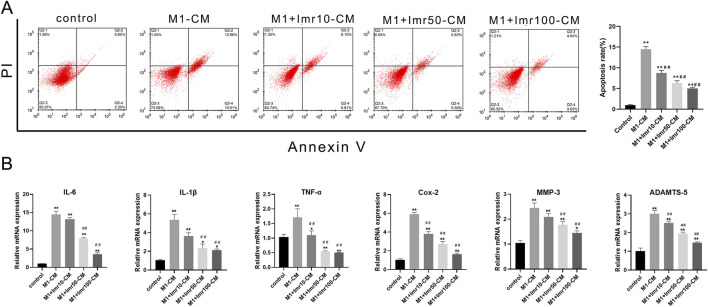
Imrecoxib prevents impairment of chondrocytes from macrophage CM. **(A)** The apoptosis rate of chondrocytes under Imrecoxib-CM. **(B)** qRT-PCR analysis of inflammatory factors (IL-6, IL-1β, TNF-α, and Cox-2) and degenerative indicators (MMP-3 and ADAMTS5). *p < 0.05, **p < 0.01 compared to the control group; ^#^p < 0.05, ^##^p < 0.01 compared with the DMM group.

To explore whether Imrecoxib could protect chondrocytes by modulating macrophage polarization, our study evaluated the impact of conditioned medium (CM) from differently polarized macrophages (including M0, M1 and M1+differerent dose of Imrecoxib, collected within 24 h) on chondrocyte apoptosis and inflammatory factor release. Compared to the M1-CM group, Imrecoxib-CM reduced the apoptosis rate of chondrocytes (Low-dose CM: 19.50% ± 1.50%; Medium-dose CM: 16.10% ± 0.85%; High-dose CM: 9.07% ± 1.90%, p < 0.01) (Figure 6A). RT-qPCR results indicated that after intervention with CM from M1 macrophages, the mRNA expression of inflammatory factors (IL-6, IL-1β, TNF-α, and Cox-2) and degenerative indicators (MMP-3 and ADAMTS5) significantly increased, while in the Imrecoxib CM group, the expression levels of these indicators decreased in a dose-dependent manner (Figure 6C). These results suggest that Imrecoxib plays important roles in inhibiting chondrocytes apoptosis, reducing inflammation and delaying degeneration through modulating macrophage polarization.

### 3.6 Imrecoxib regulates macrophage polarization through the COX-2/PGE2 pathway

**FIGURE 7:**
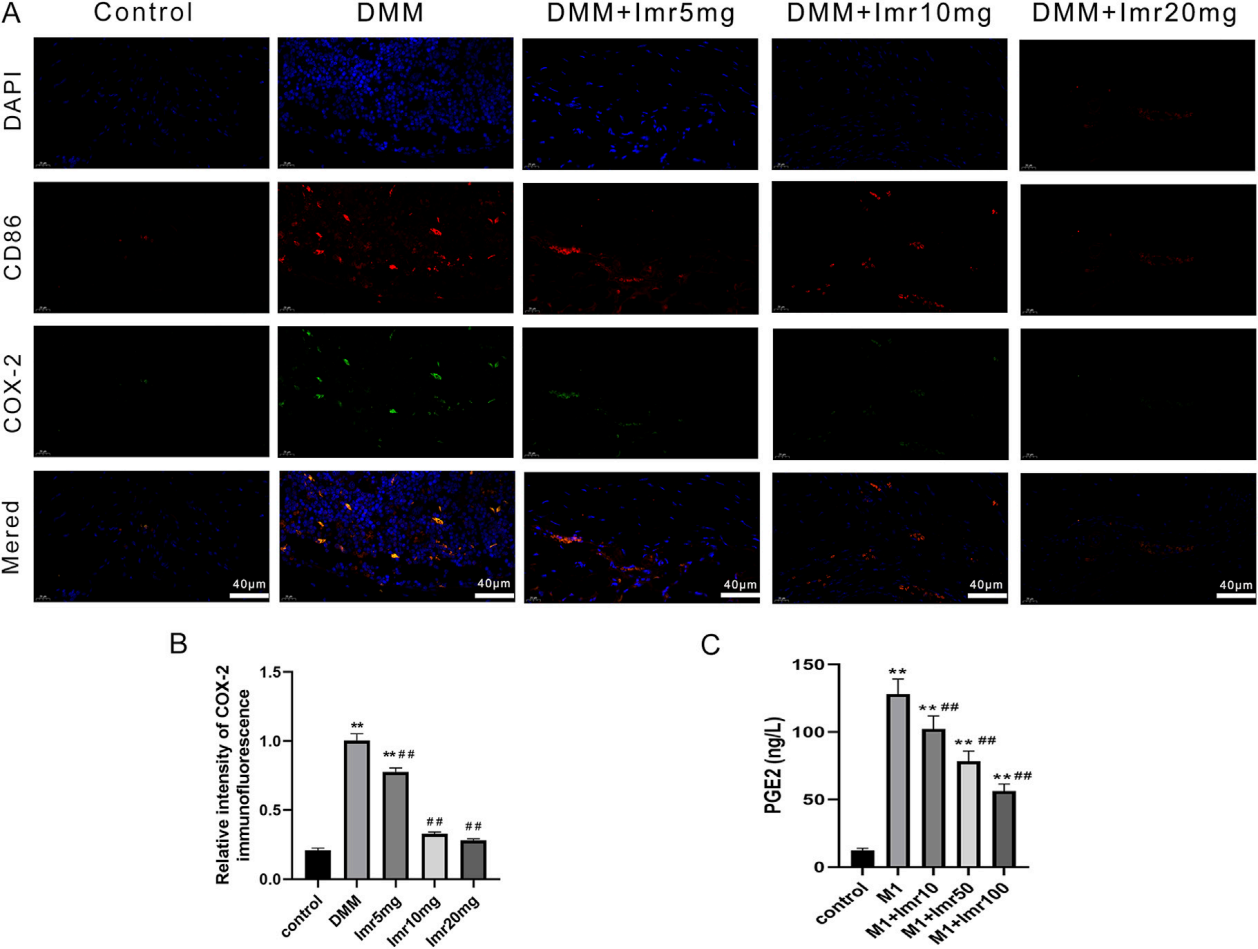
Imrecoxib regulates macrophage polarization through the COX-2/PGE2 pathway. **(A)** Immunofluorescence staining of COX-2 protein in M1 synovial macrophages. **(B)** The average fluorescence intensity was quantified. **(C)** Elisa analysis of LPS-induced secretion of PGE2 by RAW264.7 macrophages. *p < 0.05, **p < 0.01 compared to the control group; ^#^p < 0.05, ^##^p < 0.01 compared with the DMM group.

Immunofluorescence staining indicates a significant increase in COX-2 protein content in M1-type synovial macrophages. Following Imrecoxib intervention, the expression of COX-2 in M1-type synovial macrophages significantly decreased. (Figures 7A,B). After LPS stimulation, ELISA results demonstrated that Imrecoxib significantly reduced the LPS-induced secretion of PGE2 by RAW264.7 macrophages (p < 0.01) (Figure 7C). Taken together, the results suggests that Imrecoxib may modulate macrophage polarization through the COX-2/PGE2 pathway.

## 4 Discussion

Osteoarthritis (OA), a common musculoskeletal disease, is characterized by cartilage degradation and synovial inflammation, leading to joint pain, deformity, and restricted mobility (Sellam and Berenbaum, 2010). The polarization ratio of M1/M2 macrophages is positively correlated with the severity of OA (Zhang et al., 2020). Therefore, inhibiting the polarization of M1 macrophages and promoting the transition of M1 to M2 subtype may represent a novel strategy for the treatment of OA (Chen et al., 2020).

Numerous studies have indicated a strong correlation between synovitis and OA-related pain (Chan et al., 2018). Various mediators, including cytokines, proteases, neuropeptides, chemokines, and prostaglandins, are locally released in damaged tissues, triggering a series of stimuli that lead to peripheral sensitization (Sheppe et al., 2018). Imrecoxib, a commonly used nonsteroidal anti-inflammatory drug (NSAIDs), primarily exerts its analgesic effects in OA patients through selective inhibition of COX-2 (Li et al., 2020b; Liu et al., 2019). This study confirmed the alleviating effect of Imrecoxib on the osteoarthritic pain via von Frey filament and hot plate stimulation test. In the early stages of OA, bone loss is closely associated with increased bone remodeling (Hunter et al., 2013). In the contrast, there is a reduction in bone resorption and the formation of subchondral sclerosis in the late stage of OA (Krustev et al., 2015; Syx et al., 2018). Our micro-CT results indicated that Imrecoxib treatment, in a dose-dependent manner, reduced bone loss and increased the bone mass of subchondral bone, demonstrating the protective role in the integrity of the knee joint.

Further, histological staining revealed the protective effect of Imrecoxib on articular cartilage, including increased cartilage matrix and joint thickness, decreased inflammatory factors. However, in recent years, an increasing body of research indicates that macrophage polarization is a crucial checkpoint in regulating the inflammatory response in OA (Zhu et al., 2021). As a dynamic cell population, macrophages respond to stimuli in their microenvironment by modifying their phenotype and function. Hence, macrophage phenotypic changes are closely associated with functional alterations (Lluch et al., 2014). In this study, we demonstrated that Imrecoxib treatment significantly modulates synovial macrophage polarization by elevating the M2/M1 phenotypic ratio in OA joints. *In vitro* experiments further confirmed its reprogramming effects on RAW264.7 macrophage polarization, shifting the balance toward anti-inflammatory M2 phenotypes. Taken together, Imrecoxib could effectively protect joint cartilage and inhibit joint inflammation by targeting macrophage polarization, ultimately alleviating OA symptoms.

To verify whether the anti-inflammatory and anti-apoptotic effect of Imrecoxib are exerted through the regulation of macrophage polarization, conditioned medium (CM) from macrophages pretreated with Imrecoxib was utilized, which has been widely applied to investigate the crosstalk between cells (Yi et al., 2024; Ou et al., 2024). The results revealed that Imrecoxib-CM could inhibit chondrocytes apoptosis, decrease the expression levels of inflammatory factors IL-6, IL-1β, TNF-α and COX-2, as well as reduce the degenerative cytokines of MMP3 and ADAMTS5. These findings suggest that Imrecoxib exerts a protective effect on chondrocytes by modulating macrophage polarization.

Although recent research has found that some active substances attenuate osteoarthritis progression by acting on synovial macrophage polarization transformation, the underlying regulatory mechanisms remain unclear (Chen et al., 2024; Zhou et al., 2019). In addition to the classical mTOR, NF-κB, JNK, and PI3K/Akt pathway, a COX-2-dependent mechanism has also been found to modulate macrophage polarization in the development of post-incisional pain, obesity-associated insulin resistance and Hirschsprung disease-associated Enterocolitis (Meng et al., 2024; Godai et al., 2014; Chan et al., 2018). Besdies, Austin et al. found that PGE2 enhances inflammatory activation and M1 polarization in THP-1 human macrophages (Sheppe et al., 2018). Zhan et al. found that PGE2 promotes macrophage recruitment and neovascularization in murine wet-type AMD models (Zhan et al., 2022). Moreover, mesenchymal stem cells could promote type 2 macrophage polarization to ameliorate the myocardial injury via the COX-2-PGE2 pathway (Jin et al., 2019). Consistent with previous research, we found that Imrecoxib reduces the expression of COX-2 protein and the secretion of PGE2 in M1 macrophages, firstly confirming that Imrecoxib regulates synovial macrophage phenotypic polarization through the COX-2/PGE2 pathway to exert its anti-inflammatory effects.

In conclusion, our study unravels that Imrecoxib protects joint cartilage and attenuates osteoarthritis by modulating synovial macrophage polarization through inactivating COX-2/PGE2 signaling pathway, providing new drug delivery strategy for the clinical treatment of OA.

## Data Availability

The raw data supporting the conclusions of this article will be made available by the authors, without undue reservation.

## References

[B1] Chan P. Wu T. Chen Y. Lu C. Wabitsch M. Tian Y. (2018). Targeted inhibition of CD74 attenuates adipose COX-2-MIF-mediated M1 macrophage polarization and retards obesity-related adipose tissue inflammation and insulin resistance. Clin. Sci. Lond. Engl. 1979 132, 1581–1596. 10.1042/cs20180041 29773671

[B2] Chen S. Xu H. He Y. Meng C. Fan Y. Qu Y. (2024). Carveol alleviates osteoarthritis progression by acting on synovial macrophage polarization transformation: an *in vitro* and *in vivo* study. Chemico-Biological Interact. 387, 110781. 10.1016/j.cbi.2023.110781 37967808

[B3] Chen X. Bai J. Shen F. Bai A. Guo Z. Cheng G. (2004). Imrecoxib: a novel and selective cyclooxygenase 2 inhibitor with anti-inflammatory effect. Acta Pharmacol. Sin. 25, 927–931. 15210067

[B4] Chen Y. Jiang W. Yong H. He M. Yang Y. Deng Z. (2020). Macrophages in osteoarthritis: pathophysiology and therapeutics. Am. J. Transl. Res. 12, 261–268. 32051751 PMC7013211

[B5] D'Atri D. Zerrillo L. Garcia J. Oieni J. Lupu-Haber Y. Schomann T. (2021). Nanoghosts: mesenchymal Stem cells derived nanoparticles as a unique approach for cartilage regeneration. J. Control. Release 337, 472–481. 10.1016/j.jconrel.2021.05.015 34015401

[B6] Godai K. Hasegawa-Moriyama M. Kurimoto T. Saito T. Yamada T. Sato T. (2014). Peripheral administration of morphine attenuates postincisional pain by regulating macrophage polarization through COX-2-dependent pathway. Mol. pain 10, 36. 10.1186/1744-8069-10-36 24928142 PMC4079829

[B7] HüGLE T. Geurts J. (2017). What drives osteoarthritis? synovial versus subchondral bone pathology. Rheumatol. Oxf. Engl. 56, 1461–1471. 10.1093/rheumatology/kew389 28003493

[B8] Hunter D. Bierma-Zeinstra S. (2019). Osteoarthr. Lancet London, Engl. 393, 1745–1759. 10.1016/S0140-6736(19)30417-9 31034380

[B9] Hunter D. Guermazi A. Roemer F. Zhang Y. Neogi T. (2013). Structural correlates of pain in joints with osteoarthritis. Osteoarthr. Cartil. 21, 1170–1178. 10.1016/j.joca.2013.05.017 23973127

[B10] Jin L. Deng Z. Zhang J. Yang C. Liu J. Han W. (2019). Mesenchymal stem cells promote type 2 macrophage polarization to ameliorate the myocardial injury caused by diabetic cardiomyopathy. J. Transl. Med. 17, 251. 10.1186/s12967-019-1999-8 31382970 PMC6683374

[B11] Krustev E. Rioux D. Mcdougall J. (2015). Mechanisms and mediators that drive arthritis pain. Curr. Osteoporos. Rep. 13, 216–224. 10.1007/s11914-015-0275-y 26025232

[B12] Lee I. N. Stening J. Z. Rose F. R. A. J. White L. J. (2024). Functional interleukin-4 releasing microparticles impact THP-1 differentiated macrophage phenotype. Front. Bioeng. Biotechnol. 12, 1496111. 10.3389/fbioe.2024.1496111 39564101 PMC11573512

[B13] Li J. Zhang B. Liu W.-X. Lu K. Pan H. Wang T. (2020a). Metformin limits osteoarthritis development and progression through activation of AMPK signalling. Ann. Rheumatic Dis. 79, 635–645. 10.1136/annrheumdis-2019-216713 PMC7213329 32156705

[B14] Li J. Zhang H. Han Y. Hu Y. Geng Z. Su J. (2023). Targeted and responsive biomaterials in osteoarthritis. Theranostics 13, 931–954. 10.7150/thno.78639 36793867 PMC9925319

[B15] Li N. Qiao Y. Xue L. Xu S. Zhang N. (2019). Targeted and MMP-2/9 responsive peptides for the treatment of rheumatoid arthritis. Int. J. Pharm. 569, 118625. 10.1016/j.ijpharm.2019.118625 31425817

[B16] Liu B. Zhang M. Zhao J. Zheng M. Yang H. (2018). Imbalance of M1/M2 macrophages is linked to severity level of knee osteoarthritis. Exp. Ther. Med. 16, 5009–5014. 10.3892/etm.2018.6852 30546406 PMC6256852

[B17] Liu Y. Zhang R. Li Z. Zhou J. Yang T. Yang C. (2019). Lack of effect of Imrecoxib, an innovative and moderate COX-2 inhibitor, on pharmacokinetics and pharmacodynamics of warfarin in healthy volunteers. Sci. Rep. 9, 15774. 10.1038/s41598-019-51755-z 31673051 PMC6823368

[B18] Liu Z. Huang J. Wang X. Deng S. Zhou J. Gong Z. (2023). Dapagliflozin suppress endoplasmic reticulum stress mediated apoptosis of chondrocytes by activating Sirt1. Chemico-Biological Interact. 384, 110724. 10.1016/j.cbi.2023.110724 37741535

[B19] Li Y. Wang J. Huang J. Yu J. Wang Y. Tan H. (2020b). Dose investigation of imrecoxib in patients with renal insufficiency based on modelling and simulation. Eur. J. Pharm. Sci. official J. Eur. Fed. Pharm. Sci. 152, 105449. 10.1016/j.ejps.2020.105449 32621967

[B20] Lluch E. Torres R. Nijs J. VAN Oosterwijck J. (2014). Evidence for central sensitization in patients with osteoarthritis pain: a systematic literature review. Eur. J. pain 18, 1367–1375. 10.1002/j.1532-2149.2014.499.x 24700605

[B21] Manferdini C. Paolella F. Gabusi E. Gambari L. Piacentini A. Filardo G. (2017). Adipose stromal cells mediated switching of the pro-inflammatory profile of M1-like macrophages is facilitated by PGE2: *in vitro* evaluation. Osteoarthr. Cartil. 25, 1161–1171. 10.1016/j.joca.2017.01.011 28153787

[B22] Ma Y. Yang H. Zong X. Wu J. Ji X. Liu W. (2021). Artificial M2 macrophages for disease-modifying osteoarthritis therapeutics. Biomaterials 274, 120865. 10.1016/j.biomaterials.2021.120865 33991950

[B23] Meng X. Xiao J. Wang J. Sun M. Chen X. Wu L. (2024). Mesenchymal stem cells attenuates Hirschsprung diseases - associated enterocolitis by reducing M1 macrophages infiltration via COX-2 dependent mechanism. J. Pediatr. Surg. 59, 1498–1514. 10.1016/j.jpedsurg.2024.02.033 38508971

[B24] Nakata K. Hanai T. Take Y. Osada T. Tsuchiya T. Shima D. (2018). Disease-modifying effects of COX-2 selective inhibitors and non-selective NSAIDs in osteoarthritis: a systematic review. Osteoarthr. Cartil. 26, 1263–1273. 10.1016/j.joca.2018.05.021 29890262

[B25] Ou Q. Tang S. A. Zhu J. Xue S. Huang H. Zhao Y. (2024). Spermidine ameliorates osteoarthritis via altering macrophage polarization. Biochimica Biophysica Acta (BBA) - Mol. Basis Dis. 1870, 167083. 10.1016/j.bbadis.2024.167083 38367900

[B26] Roemer F. Kassim Javaid M. Guermazi A. Thomas M. Kiran A. Keen R. (2010). Anatomical distribution of synovitis in knee osteoarthritis and its association with joint effusion assessed on non-enhanced and contrast-enhanced MRI. Osteoarthr. Cartil. 18, 1269–1274. 10.1016/j.joca.2010.07.008 20691796

[B27] Scanzello C. Goldring S. (2012). The role of synovitis in osteoarthritis pathogenesis. Bone 51, 249–257. 10.1016/j.bone.2012.02.012 22387238 PMC3372675

[B28] Sellam J. Berenbaum F. (2010). The role of synovitis in pathophysiology and clinical symptoms of osteoarthritis. Nat. Rev. Rheumatol. 6, 625–635. 10.1038/nrrheum.2010.159 20924410

[B29] Sheppe A. Kummari E. Walker A. Richards A. Hui W. Lee J. (2018). PGE2 augments inflammasome activation and M1 polarization in macrophages infected with Salmonella typhimurium and Yersinia enterocolitica. Front. Microbiol. 9, 2447. 10.3389/fmicb.2018.02447 30429830 PMC6220063

[B30] Sun Y. Zuo Z. Kuang Y. (2020). An emerging target in the battle against osteoarthritis: macrophage polarization. Int. J. Mol. Sci. 21, 8513. 10.3390/ijms21228513 33198196 PMC7697192

[B31] Syx D. Tran P. Miller R. Malfait A. (2018). Peripheral mechanisms contributing to osteoarthritis pain. Curr. Rheumatol. Rep. 20, 9. 10.1007/s11926-018-0716-6 29480410 PMC6599517

[B32] Thomas E. Peat G. Croft P. (2014). Defining and mapping the person with osteoarthritis for population studies and public health. Rheumatol. Oxf. Engl. 53, 338–345. 10.1093/rheumatology/ket346 PMC3894672 24173433

[B33] Wang Y.-H. Zhu L.-L. Li T.-L. Zhou Q. (2024). Imrecoxib: advances in pharmacology and therapeutics. Drug Des. Dev. Ther. 18, 1711–1725. 10.2147/dddt.s464485 PMC11128231 38799798

[B34] Wang W. Chu Y. Zhang P. Liang Z. Fan Z. Guo X. (2023). Targeting macrophage polarization as a promising therapeutic strategy for the treatment of osteoarthritis. Int. Immunopharmacol. 116, 109790. 10.1016/j.intimp.2023.109790 36736223

[B35] Xia L. Zhang H. Xing M. Xu Y. Li P. Huang L. (2018). Knockdown of PRMT1 suppresses IL-1β-induced cartilage degradation and inflammatory responses in human chondrocytes through Gli1-mediated Hedgehog signaling pathway. Mol. Cell. Biochem. 438, 17–24. 10.1007/s11010-017-3109-7 28744817

[B36] Yang Q. Ding W. Cao Y. Zhou Y. Ni S. Shi T. (2018). Interferonregulatoryfactor-8(IRF-8) regulates the expression of matrix metalloproteinase-13 (MMP-13) in chondrocytes. Cell stress and chaperones 23, 393–398. 10.1007/s12192-017-0849-y 29247272 PMC5904082

[B37] Yi G. Zhang R. Li M. Song X. Li S. (2024). Atractylenolide-III attenuates osteoarthritis by repolarizing macrophages through inactivating TLR4/NF-κB signaling. Int. Immunopharmacol. 129, 111629. 10.1016/j.intimp.2024.111629 38346377

[B38] Zeng G. Chen A. Li W. Song J. Gao C. (2015). High MMP-1, MMP-2, and MMP-9 protein levels in osteoarthritis. Genet. Mol. Res. GMR 14, 14811–14822. 10.4238/2015.november.18.46 26600542

[B39] Zhang H. Cai D. Bai X. (2020). Macrophages regulate the progression of osteoarthritis. Osteoarthr. Cartil. 28, 555–561. 10.1016/j.joca.2020.01.007 31982565

[B40] Zhan P. Cui Y. Cao Y. Bao X. Wu M. Yang Q. (2022). PGE2 promotes macrophage recruitment and neovascularization in murine wet-type AMD models. Cell Commun. Signal. 20, 155. 10.1186/s12964-022-00973-6 36229856 PMC9558420

[B41] Zhou F. Mei J. Han X. Li H. Yang S. Wang M. (2019). Kinsenoside attenuates osteoarthritis by repolarizing macrophages through inactivating NF-κB/MAPK signaling and protecting chondrocytes. Acta Pharm. Sin. B 9, 973–985. 10.1016/j.apsb.2019.01.015 31649847 PMC6804452

[B42] Zhu X. Lee C.-W. Xu H. Wang Y.-F. Yung P. S. H. Jiang Y. (2021). Phenotypic alteration of macrophages during osteoarthritis: a systematic review. Arthritis Res. and Ther. 23, 110. 10.1186/s13075-021-02457-3 33838669 PMC8035781

